# Synergistic Effects of Nano-Sized Titanium Dioxide and Zinc on the Photosynthetic Capacity and Survival of *Anabaena* sp.

**DOI:** 10.3390/ijms140714395

**Published:** 2013-07-11

**Authors:** Yulin Tang, Shuyan Li, Junlian Qiao, Hongtao Wang, Lei Li

**Affiliations:** State Key Laboratory of Pollution Control and Resource Reuse, College of Environmental Science & Engineering, Tongji University, Shanghai 200092, China; E-Mails: shuyan198912@126.com (S.L.); qiaoqiao@tongji.edu.cn (J.Q.); wanghongtao010@126.com (H.W.); lilei@tongji.edu.cn (L.L.)

**Keywords:** synergistic toxicity, zinc, nanoparticles, titanium dioxide, *Anabaena* sp

## Abstract

*Anabaena* sp. was used to examine the toxicity of exposure to a nano-TiO_2_ suspension, Zn^2+^ solution, and mixtures of nano-TiO_2_ and Zn^2+^ suspensions. Typical chlorophyll fluorescence parameters, including effective quantum yield, photosynthetic efficiency and maximal electron transport rate, were measured by a pulse-amplitude modulated fluorometer. Nano-TiO_2_ particles exhibited no significant toxicity at concentrations lower than 10.0 mg/L. The 96 h concentration for the 50% maximal effect (EC_50_) of Zn^2+^ alone to *Anabaena* sp. was 0.38 ± 0.004 mg/L. The presence of nano-TiO_2_ at low concentrations (<1.0 mg/L) significantly enhanced the toxicity of Zn^2+^ and consequently reduced the EC_50_ value to 0.29 ± 0.003 mg/L. However, the toxicity of the Zn^2+^/TiO_2_ system decreased with increasing nano-TiO_2_ concentration because of the substantial adsorption of Zn^2+^ by nano-TiO_2_. The toxicity curve of the Zn^2+^/TiO_2_ system as a function of incremental nano-TiO_2_ concentrations was parabolic. The toxicity significantly increased at the initial stage, reached its maximum, and then decreased with increasing nano-TiO_2_ concentration. Hydrodynamic sizes, concentration of nano-TiO_2_ and Zn^2+^ loaded nano-TiO_2_ were the main parameters for synergistic toxicity.

## 1. Introduction

Heavy metals are discharged into aquatic ecosystems from various industries, such as the textile, mining, electroplating, and metallurgical industries. Heavy metals are persistent environmental contaminants that cannot be destroyed or degraded [1]. Heavy metals pose a serious threat to human beings and aquatic ecosystems because of their persistent toxicity, bioaccumulation, and biomagnifications through the food chain. Algae, a class of organisms forming the basic nourishment for the food chain, are commonly used as model organisms to study the toxicity of heavy metals [2]. Recent studies have focused on the interaction between heavy metals and different aquatic conditions, such as temperature, irradiance, pH, ethylenediaminetetraacetic acid (EDTA), anions, and nutrients in algae [3,4].

The expansion of nanotechnology has resulted in subsequent increased release of nanoparticles (NPs) into aquatic environments during the cycle of manufacturing, transportation, consumption, and disposal [5]. Among these NPs, nano-sized titanium dioxide (nano-TiO_2_) is one of the most popular engineered nanomaterials increasingly being incorporated into various consumer products. The negative environmental effects of NPs have drawn significant attention in recent years [6–8]. Numerous studies have focused on the inhibitory effects of titanium dioxide, zinc oxide, copper oxide, silicon oxide, and alumina NPs in algae [9–12]. Scholars have obtained different results regarding the toxicity mechanism of oxide NPs to algae, such as the contribution of dissolved metal ions from NPs or the agglomerates of NPs onto algae [10–13].

Heavy metals including Zn, Cd, Pb, Ni, Cu, and Co have adverse effects on the growth, cell division, photosynthesis, and destruction of primary metabolites in algae [14–18]. The toxicity of heavy metals is usually a function of free heavy metal ions because these species are generally the most bioavailable ones [4,19]. This toxicity is likely associated with glutathione redox cycle, reactive oxygen species production, and phytohormone production [18,20,21]. The individual toxicity of NPs and heavy metals to algae has been widely investigated [10–13,16–19]. Studies on the synergistic effects of these two categories on algae are limited and controversial. The toxicity of heavy metals to green algae is eliminated in the presence of TiO_2_ NPs with high surface area [22,23]. However, the inhibition of green algae at the same heavy metal concentration is not notably affected by adding various sizes of TiO_2_ NPs [24].

To further explore the mechanism of the synergistic toxicity of NPs and heavy metals to algae, we investigate how TiO_2_ NP_S_ influence the bioavailability of heavy metal zinc (Zn). Zn is an essential component of various enzymes for algae, particularly those in photosynthetic electron transport. At elevated concentrations, Zn is toxic with its most toxic form Zn^2+^[25]. Algae and cyanobacteria are abundant in aquatic ecosystems and envisaged as an ideal model to study any adverse effects of released NPs [26]. The cyanobacterium *Anabaena* sp. is used as a model to study the toxicity of nano-TiO_2_ and Zn^2+^. Current use of algae in the study of NPs toxic effects on photosynthesis seems to be a convenient method [27]. The change of photosynthetic activity affects the photosynthetic process and cellular growth, which may be indicated by fluorescence emission. Fluorescence measurements thus serve as an important indicator to provide information of NPs interaction with photosynthesis and toxic effects on the physiological state of algae [28].

The objective of this study is to determine the synergistic toxicity of nano-TiO_2_ and Zn^2+^ on *Anabaena* sp. using a pulse-amplitude modulated (PAM) fluorometer, a rapid and efficient tool for *in vivo* studies of photosynthetic activity. The correlation between algal cell growth and photosynthetic fluorescence parameters of soluble Zn^2+^ alone and nano-TiO_2_ on *Anabaena* sp. is also investigated to provide background information for toxicity comparisons.

## 2. Results and Discussion

### 2.1. Characterization and Sedimentation of Nano-TiO_2_ in Culture Medium

**Figure 1:**
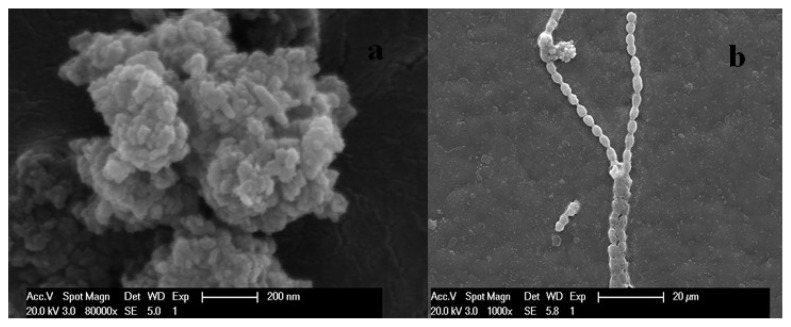
SEM images of (**a**) nano-TiO_2_; (**b**) algae in the presence of 1.0 mg/L nano-TiO_2_.

In this study, the nominal diameter of commercial nano-TiO_2_ ranged from 40 to 50 nm. As shown in the dynamic light scatting (DLS) results and Scanning electron microscope (SEM) images in Figure 1a, the average diameter of the NPs suspended in BG11 culture medium dramatically increased to approximately 450 to 650 nm. The aggregation tendency of the NPs was ascribed to the relatively low zeta potential (−7.8 mV). This rapid formation of nano-TiO_2_ aggregates was also observed in previous studies [29,30], indicating that algae and other living organisms were exposed to nano-TiO_2_ beyond their original nanoscale particle size in environment systems. At the same time, aggregated nano-TiO_2_ were much more toxic than their bulk counterpart [31]. The nano-TiO_2_ attached on the surface of the algal cells and the direct contact is demonstrated clearly by the SEM images in Figure 1b. So, the hydrodynamic size and the adsorption of nano-TiO_2_ on algae affected their toxicity.

### 2.2. Sorption of Zn^2+^ onto Nano-TiO_2_

**Figure 2:**
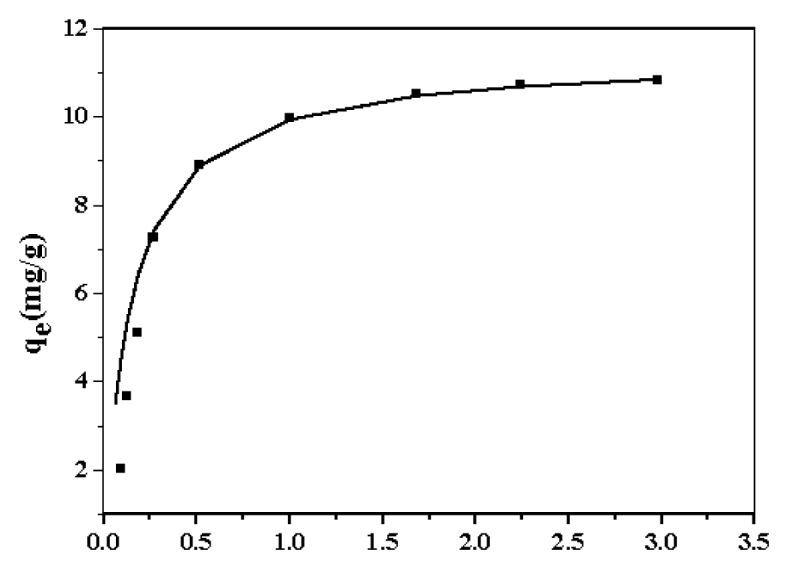
Adsorption isotherms of Zn^2+^ on nano-TiO_2_ in the culture medium; pH = 7.0; temperature = 298 K.

The interactions of Zn*^2+^* with nano-TiO_2_ were determined by examining the sorption equilibrium. In the equilibrium isotherm experiment, a correlation between Zn^2+^ adsorbed on the nano-TiO_2_ (*q*_e_, mg/g) and the non-adsorbed Zn^2+^ concentration (*C*_e_, mg/L) in the culture medium was determined. Figure 2 shows the sorption density of nano-TiO_2_ as a function of initial Zn^2+^ concentration up to 10.0 mg/L. Nano-TiO_2_ could adsorb Zn^2+^ from the culture medium. With the initial Zn^2+^ concentration increased from 3.0 to 10.0 mg/L, Zn^2+^ adsorption approached the saturation point. The Langmuir isotherm was used to fit these adsorption data using Matlab.

(1) $$\frac{C_{e}}{q_{e}}=\frac{C_{e}}{q_{\text{max}}}+\frac{1}{q_{\text{max}}b}$$

The calculated adsorption capacity *q*_max_ was approximately 11.38 mg/g, and the parameter for the *b* = 6.92. A good correlation is shown in Figure 2 suggesting a monolayer adsorption of Zn^2+^ on nano-TiO_2_. Therefore, nano-TiO_2_ adsorption had an important impact on the Zn^2+^ concentration in the culture medium.

### 2.3. Toxicity of Nano-TiO_2_

**Figure 3:**
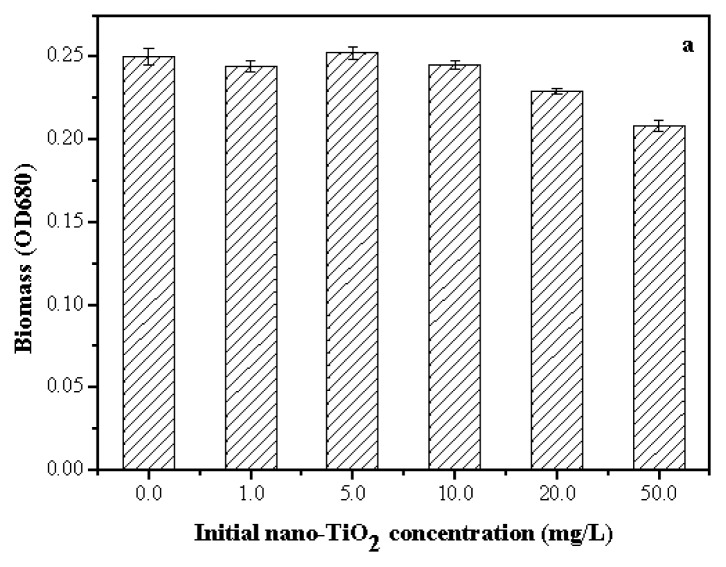

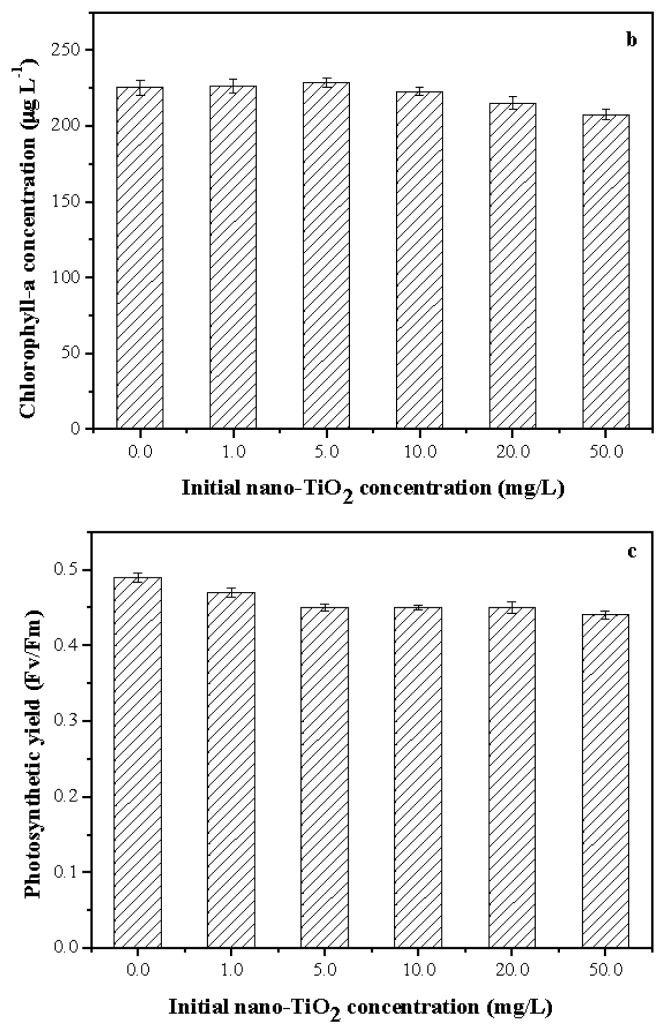
Toxic effect of nano-TiO_2_ on the inhibition of *Anabaena* sp. at 96 h. (**a**) Biomass of algae at different initial level of nano-TiO_2_; (**b**) Chlorophyll-a concentration at different level of nano-TiO_2_; (**c**) Photosynthetic yield of algae at different initial level of nano-TiO_2_.

The toxicity of nano-TiO_2_ to algae was reported by other researchers [11,22,23]. However, these results were not significantly comparable because of the different sources and properties of NPs. The inhibition of *Anabaena* sp. at different nano-TiO_2_ concentrations from 1.0 to 50.0 mg/L is shown in Figure 3. After 96 h of exposure, the inhibition was observed at nano-TiO_2_ concentrations more than 10.0 mg/L to algae. The changes in the content of chlorophyll-a and the photochemical transformation of energy were observed. The difference in the toxicity of nano-TiO_2_ could be related to particle size, crystal form, and test method. Large aggregates of TiO_2_ nanoparticles entrapped algal cells (Figure 1b), which reduced the light available to the algal cells and inhibited their growth [32,33]. Moreover, nutrients adsorbed by nano-TiO_2_ in culture medium would contribute to the toxicity [34].

### 2.4. Toxicity of Zn^2+^ in the Absence and Presence of Nano-TiO_2_

**Figure 4:**
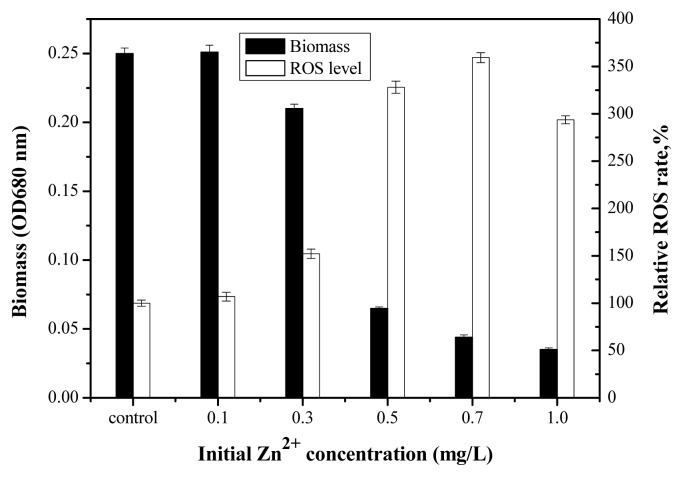
Inhibition of *Anabaena* sp. growth and relative ROS rate at different initial concentrations of Zn^2+^.

**Figure 5:**
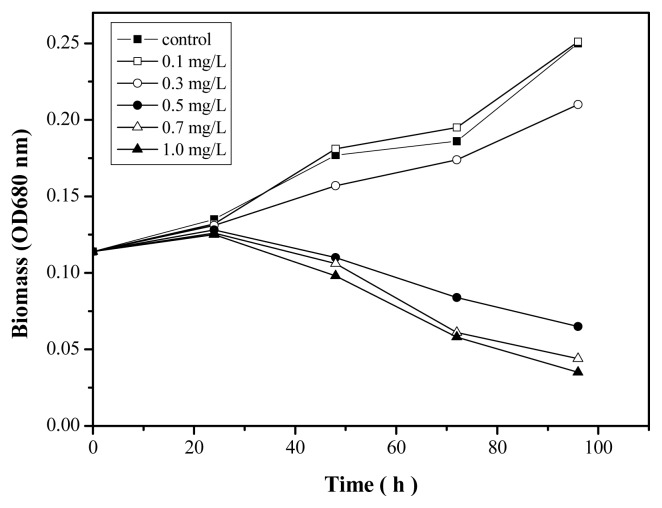
The growth process of *Anabaena* sp. at different initial concentrations of Zn^2+^.

Figure 4 shows the inhibition of *Anabaena* sp. at different Zn^2+^ concentrations after 96 h. No significant inhibition was observed at Zn^2+^ concentrations below 0.3 mg/L, whereas the biomass of *Anabaena* sp. notably decreased with increasing Zn^2+^ concentration from 0.5 to 1.0 mg/L. The 96 h growth process of *Anabaena* sp. with increasing Zn^2+^ concentration from 0 to 1.0 mg/L is shown in Figure 5. The exposure of *Anabaena* sp. to Zn^2+^ resulted in a clear difference in cell number between the control and experimental samples. Higher initial Zn^2+^ concentrations reduced cell density significantly. Growth inhibition was essentially proportional to Zn^2+^ concentration. However, at the lowest Zn^2+^ concentration considered (0.1 mg/L), an increase in the growth of *Anabaena* sp. was actually observed. The 96 h EC_50_ value for *Anabaena* sp. growth was calculated to be 0.38 ± 0.004 mg/L. This finding was in accordance with the results of a previous study on the exposure of *Micractinium pusillum* to Zn [35]. When Zn^2+^ was in high concentration, *Anabaena* sp. created physiological stress leading to generation of free radicals. Stress in turn induced the production of reactive oxygen species (ROS). The ROS could rapidly attack all types of biomolecules such as nucleic acids, protein, lipids, and amino acids, leading to irreparable metabolic dysfunction and algae death [36]. Results from H_2_DCF-DA dye test using microplate reader showed that the intracellular ROS was raised in the algal cells with different initial Zn^2+^ concentration in Figure 4. When the initial Zn^2+^ concentration was higher than 0.7 mg/L, the intracellular ROS entered in the medium with the algal cells rupture.

**Figure 6:**
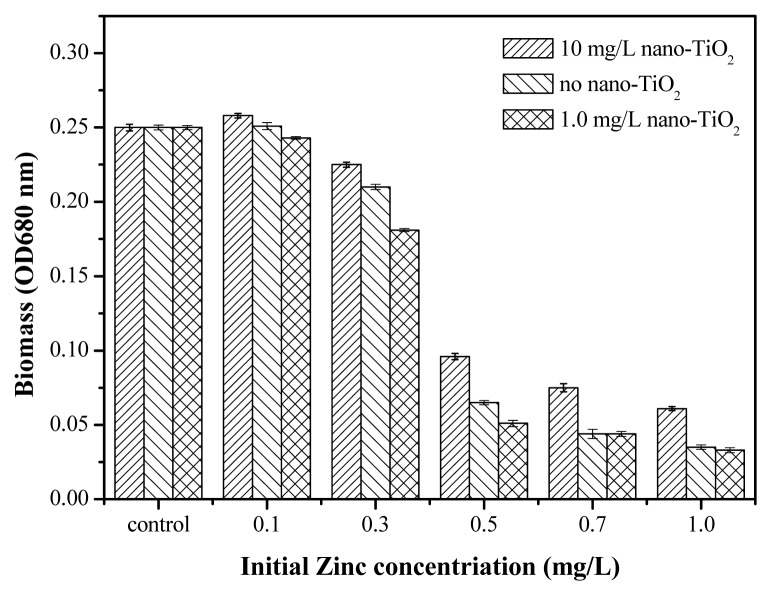
Toxic effect of Zn^2+^ on the inhibition of *Anabaena* sp. with the fixed nano-TiO_2_ at 96 h.

The synergistic toxic effect of Zn^2+^ and nano-TiO_2_ was examined using a fixed concentration of nanoparticles; the nano-TiO_2_ particles alone were not toxic at low concentrations from 1.0 to 10.0 mg/L. Figure 6 shows the effect of Zn^2+^ on the 96 h growth process of *Anabaena* sp. in the presence of nano-TiO_2_. The nanoparticles significantly impacted the toxicity of Zn^2+^. At high concentration such as 10.0 mg/L, the toxicity of Zn^2+^ was reduced and the EC_50_ value of Zn^2+^ was 0.49 ± 0.001 mg/L. A high nano-TiO_2_ concentration could effectively reduce the soluble Zn^2+^ by adsorbing Zn^2+^ on NP_S_ in Table S1. At same time, as shown in Figure S2, nano-TiO_2_ at high concentrations easily settled to the bottom of the reactor, so the soluble Zn^2+^ concentration around algae was low and the toxicity was reduced. At low concentration such as 1.0 mg/L, the toxicity of Zn^2+^ was enhanced and the EC_50_ value of Zn^2+^ with 1.0 mg/L nano-TiO_2_ was about 0.29 ± 0.005 mg/L. The results indicate that Zn^2+^ toxicity was significantly enhanced by 1.0 mg/L nano-TiO_2_ in the culture medium. However, the low concentration of nano-TiO_2_ reduced the soluble Zn^2+^ concentration, as shown in Table S1. Soluble Zn^2+^ and adsorbed Zn^2+^ were believed to contribute to the overall toxic effect on algae. The direct adherence of nano-TiO_2_ resulted in a high localized concentration on the algal surface, which could be due to high levels of free Zn^2+^[37]. Nano-TiO_2_ at low concentrations was relatively stable in the culture medium. Nano-TiO_2_ easily attached on the surface of the algal cells, which limited their mobility. The adsorbed Zn^2+^ had direct contact with the algae. The synergistic toxic effect of Zn^2+^ and nano-TiO_2_ was attributed to the concentration of nano-TiO_2_ and the free Zn^2+^.

### 2.5. Toxicity of Nano-TiO_2_ in the Presence of Zn^2+^

**Figure 7:**
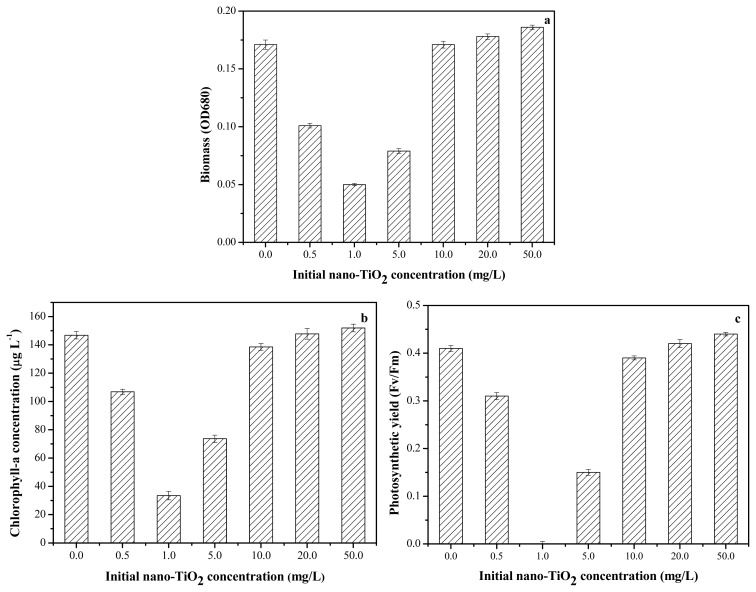
Toxic effect of Nano-TiO_2_ on the inhibition of *Anabaena* sp. with the fixed Zn^2+^ at 96 h. (**a**) Biomass of algae at different initial level of nano-TiO_2_; (**b**) Chlorophyll-a concentration at different level of nano-TiO_2_; (**c**) Photosynthetic yield of algae at different initial level of nano-TiO_2_.

Although Zn^2+^ at concentrations below 0.3 mg/L showed no significant toxic effects on *Anabaena* sp., a synergistic effect might occur if nano-TiO_2_ was also present in this system. The toxicity of nano-TiO_2_ in the presence of constant concentrations of Zn^2+^ was examined. Figure 7 shows the nano-TiO_2_ toxicity result after 96 h of exposure at 0.3 mg/L Zn^2+^. It could be seen that the toxicity of nano-TiO_2_ in the presence of Zn^2+^ was significantly different from that of nano-TiO_2_ alone. With increasing nano-TiO_2_ concentration, the inhibition of *Anabaena* sp. increased at the initial stage and then decreased afterwards. The same trend was observed in the photochemical transformation of energy and in the chlorophyll content of *Anabaena* sp. When the added nano-TiO_2_ was more than 1.0 mg/L, the overall toxicity decreased. This could have been caused by the adsorption of Zn^2+^ onto the nano-TiO_2_, which significantly reduced the soluble Zn^2+^ concentration with high concentration of nano-TiO_2_. Increased nano-TiO_2_ enhanced aggregation, resulting in a lower suspended concentration. Thus, the overall toxicity could also be decreased by reduced uptake of nano-TiO_2_ by algae [37]. The addition of nano-TiO_2_ enhanced Zn^2+^ toxicity, with the maximum enhancement observed at 1.0 mg/L nano-TiO_2_. This result is consistent with the results shown in Figure 6. By contrast, nano-TiO_2_ was non-toxic at concentrations less than 1.0 mg/L, and the contribution of bare nano-TiO_2_ to algal toxicity was neglected. The soluble Zn^2+^ concentration decreased in the presence of nano-TiO_2_, however, the overall toxicity significantly increased. First, the decrease in residual Zn^2+^ concentration reduced the toxic effect. This result was similar to the scenario for both heavy metals and other carriers [38]. Second, the adsorbed Zn^2+^ on nano-TiO_2_ contributed to toxicity once nano-TiO_2_ was taken up by algae. The addition of nano-TiO_2_ increased the total uptake of Zn^2+^-loaded nanoparticles, and the mortality increased accordingly.

NPs in aquatic systems produced potential risks, not only from nano-particles, but also from their ability to accumulate and enhance the toxicity of these background contaminants. Nano-TiO_2_ alone at low concentrations (<10.0 mg/L) did not cause significant inhibitory effects. Thus, its fate and potential aquatic effects could be easily overlooked. However, low-concentration nano-TiO_2_ served as Zn^2+^ carriers and increased the total Zn^2+^ uptake by algae. Moreover, the concentrations of the NPs in the water body were always at the microgram level. The biomagnifications of NPs from lower trophic aquatic organisms to higher ones strengthened this risk [6]. Therefore, the synergistic effects of the background toxic substances with released NPs could be more serious than the effects of NP alone.

## 3. Experimental Section

### 3.1. Culture of *Anabaena* sp

Samples of *Anabaena* sp. were obtained from the Institute of Wuhan Hydrobiology (China). The composition of BG11 culture medium is listed in Table S2. NaNO_3_, K_2_HPO_4_, MgSO_4_·7H_2_O, CaCl_2_·2H_2_O, citric acid, ferric ammonium citrate, EDTANa_2_, Na_2_CO_3_, H_3_BO_3_, MnCl_2_·4H_2_O, Na_2_MoO_4_, CuSO_4_·5H_2_O, Co(NO_3_)_2_, and ZnSO_4_·7H_2_O were purchased from Sinopharm Medicine. The deionized water (DI) used to prepare reagents and culture medium was purified by Millipore reverse osmosis. The initial pH of the medium was adjusted to 7.0 using 0.01 M HCl or NaOH solution. The algae were produced by cultivation in a constant-temperature incubator at 25 ± 1 °C. The illumination intensity in the incubator was 4000 Lux with a light-dark cycle of 12 h:12 h. The stock culture of *Anabaena* sp. were shaken three to four times a day, and their growth curves were recorded to ensure that the algae used in the test were in the logarithmic growth phase. The concentrations of experimental samples were measured using a spectrophotometer. The optical density (OD) values were in linear relation to algal concentration. The OD at 680 nm of the algal culture was 0.11 to 0.12, which corresponded to an algal concentration of 2.32 × 10^9^ cells/L.

### 3.2. Characterization and Behavior of Nano-TiO_2_ in the Medium

Nano-TiO_2_ particles (rutile form) 40 to 50 nm in diameter were purchased from Zhejiang Hongsheng Material Technology Co., China. The suspensions (1000 mg/L) were placed in an ultra-sound water bath (100 W, 40 kHz) for 30 min before being diluted to different exposure concentrations. Zeta potentials and particle sizes of nano-TiO_2_ were measured by a dynamic light scatting (DLS) size analyzer (Zetasizer Nano-ZS, Malvern, UK). Scanning electron microscope (SEM) images were taken using a JEOL SM4800 SEM. Suspensions of 1.0 and 10.0 mg/L nano-TiO_2_ were prepared by dilution in the culture medium. At 10 min intervals, the absorbance of nano-TiO_2_ suspension was measured using a UV-vis spectrophotometer. The settling behavior of the NPs was investigated by the reduction of absorbance over 600 min.

### 3.3. Sorption of Zn^2+^ on Nano-TiO_2_

The interactions of Zn^2+^ with nano-TiO_2_ were studied by performing the traditional batch sorption experiment. A stock solution of Zn^2+^ was prepared by dissolving ZnSO_4_ into DI water. The solution was diluted into 125 mL flasks to serial concentrations of 0.1, 0.2, 0.4, 0.5, 1.0, 2.0, 3.0, 5.0, and 10.0 mg/L with 50 mL culture medium. The pH of the Zn^2+^ solutions was adjusted to 7.0 ± 0.1 using 0.01 M HCl or NaOH. The nano-TiO_2_ suspensions were diluted to a concentration of 10.0 ± 0.1 mg/L in each flask. The mixed suspensions were then shaken to achieve sorption equilibrium within 10 h. The mixed suspensions were centrifuged at 5000 rpm for 10 min. The supernatants were collected and again centrifuged at 5000 rpm for 10 min [9]. Zinc concentrations in the supernatants were measured by inductively coupled plasma atomic emission spectroscopy (ICP-optima 2001DV, Perkin-Elmer, Waltham, MA, USA).

### 3.4. Toxicity Tests

In the toxicity tests, the algal growth results were obtained by the difference between the final and initial algae densities and chlorophyll fluorescence parameters. Growth of *Anabaena* sp. density was monitored daily for 96 h and assessed by initial and final OD value at 680 nm. All chlorophyll fluorescence parameters were determined using a Phyto-PAM fluorometer (Pyhto-PAM, Walz, Germany). Phyto-PAM is a four-wavelength chlorophyll fluorometer used to assess the chlorophyll content and photosynthetic activity of planktonic algae. The variables Chl-*a* fluorescence (*F*_v_) and maximal fluorescence (*F*_M_) were measured. Photosystem II activity was determined using the Δ*F* mode (*F*, fluorescence yield = *F*_M_ − *F*_v_). The yield (*Y*, photochemical transformed energy) was calculated as *Y = F*_v_*/F*_M_. In recent years, fluorescence parameters based on fluorescence yield have been proposed to be a useful tool for the toxic evaluation of pollutants [28].

The toxicity experiments were carried out using 50 mL cultures grown in 125 mL flasks. The *Anabaena* sp. solutions with a series of Zn^2+^ concentrations were cultured and observed in an incubator. The final and initial algae densities and chlorophyll fluorescence parameters were used to examine Zn^2+^ toxicity. The *Anabaena* sp. solutions with a series of nano-TiO_2_ concentrations were tested following the same methods in Zn^2+^ toxicity tests. To investigate the synergistic effects of Zn^2+^ and nano-TiO_2_, two sets of experiments were studied. The first set of experiments studied the toxic effects of Zn^2+^ with fixed nano-TiO_2_ concentrations. The second set of experiments examined the toxic effects of nano-TiO_2_ with fixed Zn^2+^ concentrations. After the toxicity test, the mixed suspensions were centrifuged and the supernatants were collected. Zinc concentrations in the supernatants were measured by ICP.

ROS production was measured by using the cell permeable indicator 2′,7′-dichlorodihydrofluorescein diacetate (H_2_DCF-DA) [39]. The specific method of operation is that 1.0 mL algal cells grown for 72 h were centrifuged at 10,000 rpm for 10 min, after which the supernatant was discarded, washed with phosphate buffer solution twice, followed immediately by the addition of 10 μM H_2_DCF-DA to the cell pellet. Next they were incubated in a water bath at 37 °C for 2 h in the dark, and washed with PBS again. The fluorescence intensity of algae cells was measured by a fluorescence microplate reader (Synergy™, Bio-Tek, Richmond, CA, USA) at excitation/emission wavelengths of 488/525 nm. Changes in ROS levels as compared to the control were evaluated using relative ROS level.

### 3.5. Statistical Analysis

The effective concentrations causing 50% inhibition in algal growth (EC_50_) were calculated and statistical significance was considered at the *p* < 0.05 level. Differences in growth rates between the control and experimental samples were demonstrated using a comparison of means test for each test concentration. Algal toxicity tests with Anabaena sp. were performed in triplicate. Data were presented as the average values of three parallel detections.

## 4. Conclusions

The mortality of *Anabaena* sp. was mostly a result of Zn^2+^ uptake. At a fixed nano-TiO_2_ concentration, the mortality was also dependent on Zn^2+^ concentration. However, at a fixed Zn^2+^ concentration, the addition of nano-TiO_2_ had a dual effect on *Anabaena* sp. At low nano-TiO_2_ concentrations, the mortality increased with increasing nano-TiO_2_. When the nano-TiO_2_ concentration reached a certain value, the amount of Zn^2+^ dissolved and adsorbed by algae sharply decreased. High nano-TiO_2_ concentrations reduced aggregation, which decreased the mortality of *Anabaena* sp. with increasing nano-TiO_2_. The results revealed that photosynthetic parameters were useful in predicting the synergistic toxicity profiles of NPs and heavy metals.

## Supplementary Information



## References

[1] Bulgariu D., Bulgariu L. (2012). Equilibrium and kinetics studies of heavy metal ions biosorption on green algae waste biomass. Bioresour. Technol.

[2] Silva A., Figueiredo S.A. (2009). Ecotoxicity tests using the green algae *Chlorella vulgaris*—A useful tool in hazardous effluents management. J. Hazard. Mater.

[3] Ismael R.P., Coral G.G. (2009). Metal toxicity to *Chlorella pyrenoidosa* assessed by a short-term continuous test. Toxicology.

[4] Miao A.J., Wang W.X. (2006). Toxicity and bioaccumulation of copper in three green microalgae species. Aquat. Toxicol.

[5] Yon J.-N., Jamie L.R. (2008). Manufactured nanoparticles: An overview of their chemistry, interactions and potential environmental implications. Sci. Total Environ.

[6] Zhu X.S., Zhou J., Cai Z.H. (2011). Suppression of chlorella vulgaris growth by cadmium, lead, and copper stress and its restoration by endogenous brassinolide. Environ. Sci. Technol.

[7] Hu J., Wang D.M., Wang J.T., Wang J.M. (2012). Phytohormones as regulators of heavy metal biosorption and toxicity in green alga *Chlorella vulgaris* (Chlorophyceae). Environ. Pollut..

[8] Oukarroum A., Bras S., Perreault F., Popovic R. (2012). Cadmium toxicity to two marine phytoplankton under different nutrient conditions. Ecotoxicol. Environ. Saf.

[9] Ji J., Long Z.F., Lin D.H. (2011). Znic acclimation and its effect on the Znic tolerance of Raphidocelis Subcapitata and *Chlorella vulgaris* in laboratory experiments. Chem. Eng. J.

[10] Levonas M., Celine C., Louis F. (2011). Evaluation of the role of the glutathione redox cycle in Cu (II) toxicity to green algae by a chiral perturbation approach. Environ. Toxicol. Chem.

[11] Metzler D.M., Li M.H. (2011). Heavy metal-induced oxidative stress in algae. Chem. Eng. J.

[12] Sadiq I.M., Pakrashi S., Chandrasekaran N., Mukherjee A. (2011). Effect of pH, EDTA, and anions on heavy metal toxicity toward a bioluminescent cyanobacterial bioreporter. J. Nanopart. Res.

[13] Lin D.H., Ji J., Tian X.L., Liu N., Yang K., Zhu L.Z., Xing B.S., Wu F.C., Wang Z.Y. (2009). Temperature and irradiance influences on cadmium and zinc uptake and toxicity in a freshwater cyanobacterium, *Microcystis aeruginosa*. Chin. Sci. Bull.

[14] Yan H., Pan G. (2002). Bioaccumulation of Fe_2_O_3_(magnetic) nanoparticles in *Ceriodaphnia dubia*. Chemosphere.

[15] Lin K.C., Lee Y.L., Chen C.Y. (2007). Inhibitory effects of silver nanoparticles in two green algae, *Chlorella vulgaris* and *Dunaliella tertiolecta*. J. Hazard. Mater.

[16] Zeng J., Yang L.Y., Wang W.X. (2009). TiO_2_ nanoparticles in the marine environment: Impact on the toxicity of tributyltin to abalone (*Haliotis diversicolor* supertexta) embryos. Aquat. Toxicol.

[17] Bajguz A. (2011). Toxicity of oxide nanoparticles to the green algae Chlorella sp. Arch. Environ. Contam. Toxicol.

[18] Alicja P.N., Andrzej B. (2012). Toxicity of copper oxide nanoparticle suspensions to aquatic biota. Plant Physiol. Biochem.

[19] Muyssen B.T.A., Janssen C.R. (2001). Responses of algae to photocatalytic nano-TiO_2_ particles with an emphasis on the effect of particle size. Chemosphere.

[20] Pinto E. (2003). Studies on toxicity of aluminum oxide (Al_2_O_3_) nanoparticles to microalgae species: *Scenedesmus* sp. and *Chlorella* sp. J. Phycol.

[21] Chen H., Chen J., Guo Y.N. (2012). Evaluation of the role of the glutathione redox cycle in Cu(II) toxicity to green algae by a chiral perturbation approach. Aquat. Toxicol..

[22] Yang W.W., Miao A.J., Yang L.Y. (2012). Cd^2+^ toxicity to a Green Alga *Chlamydomonas reinhardtii* as influenced by its adsorption on TiO_2_ engineered nanoparticles. PLoS One.

[23] Yang W.W., Li Y., Miao A.J., Yang L.Y. (2012). Cd^2+^ toxicity as affected by bare TiO_2_ nanoparticle sand their bulk counterpart. Ecotoxicol. Environ. Saf.

[24] Hartmann N.B., Kammer F.V., Hofmann T., Baalousha M., Ottofuelling S., Baun A. (2010). Algal testing of titanium dioxide nanoparticles-Testing considerations, inhibitory effects and modification of cadmium bioavailability. Toxicology.

[25] Baumann A., Morrison L., Stengel D.B. (2009). Metal accumulation and toxicity measured by PAM-Chlorophyll fluorescence in seven species of marine macroalgae. Ecotoxicol. Environ. Saf.

[26] Rodea P.I., Gonzalo S., Santiago M.J., Leganes F., Garcia C.E., Rosal R., Fernandez P.F. (2012). An insight into the mechanisms of nanoceria toxicity in aquatic photosynthetic organisms. Aquat. Toxicol..

[27] Juhel G., Batisse E., Hugues Q., Daly D. (2011). Alumina nanoparticles enhance growth of Lemna minor. Aquat. Toxicol.

[28] Dewez D., Didur O., Vincent H., Popovic R. (2008). Validation of photo synthetic-fluorescence parameters as biomarkers for isoproturon toxic effect on alga *Scenedesmus obliquus*. Environ. Pollut.

[29] French R.A., Jacobson A.R., Kim B., Isley S.L., Penn R.L., Baveye P.C. (2009). Influence of ionic strength, pH, and cation valence on aggregation kinetics of titanium dioxide nanoparticles. Environ. Sci. Technol.

[30] Keller A.A., Wang H.T., Zhou D.X., Lenihan H.S., Cherr G., Cardinale B.J., Miller R., Ji Z.X. (2010). Stability and aggregation of metal oxide nanoparticles in natural aqueous matrices. Environ. Sci. Technol.

[31] Aruoja V., Dubourguier H.-C., Kasemets K., Kahru A. (2009). Toxicity of nanoparticles of CuO, ZnO and TiO_2_ to microalgae *Pseudokirchneriella subcapitata*. Sci. Total Environ.

[32] Huang C.P. (2011). Responses of algae to photocatalytic nano-TiO_2_ particles with an emphasis on the effect of particle size. Chem. Eng. J.

[33] Wiesner M.R., Loway G.V. (2006). Accessing the Risk of Manufactured Nanomaterials.

[34] Kuwabara J.S., Davis J.A., Chang C.C.Y. (1986). Algal growth response to particle-bound orthophosphate and zinc. Limnol. Oceanogr.

[35] Toumi A., Belkoura M., Benabdallah S., Alami M., Idrissi L., Nejmeddine A. (2007). Effect and bioaccumulation of heavy metals (Zn, Cd) on Micractinium pusillum Alga. Environ. Technol.

[36] Choudhary M., Jetley U.K., Khan M.A. (2007). Effect of heavy metal stress on proline, malondialdehyde, and superoxide dismutase activity in the cyanobacterium *Spirulina platensis-*S5. Ecotoxicol. Environ. Saf.

[37] Zhu X.S., Tian S.Y., Cai Z.H. (2012). Toxicity assessment of iron oxide nanoparticles in zebrafish early life stages. PLoS One.

[38] Wang D.M., Hu J., Irons D.R. (2011). Synergistic toxic effect of nano-TiO_2_ and As(V) on *Ceriodaphnia dubia*. Sci. Total Environ.

[39] Qian H.F., Li J.J., Sun L.W. (2009). Combined effect of copper and cadmium on *Chlorella vulgaris* growth and photosynthesis-related gene transcription. Aquat. Toxicol.

