# Supplementary material for: Synergistic Effects of Nano-Sized Titanium Dioxide and Zinc on the Photosynthetic Capacity and Survival of Anabaena sp
Source: Int J Mol Sci. 2013 Jul 11;14(7):14395–407. doi: 10.3390/ijms140714395 (PMC3742250; doi:10.3390/ijms140714395)
Supplement: Supplementary file 1 [file ijms-14-14395-s001.pdf]

Supplementary Information

Table S1. The remaining Zn^{2+} concentration in the supernatants of the algae solution.

Initial Zn^{2+} concentration (mg/L)	The remaining Zn^{2+} concentration (mg/L)		
	***Anabaena* sp.**	***Anabaena* sp./TiO_2 (1.0 mg/L)**	***Anabaena* sp./TiO_2 (10.0 mg/L)**
0.1	0.048	0.045	0.044
0.3	0.126	0.093	0.085
0.5	0.249	0.198	0.188
0.7	0.410	0.341	0.222
1.0	0.538	0.440	0.331

Table S2. Compounds and their concentrations in the BG-11 culture medium.

Component	Amount (mL)	Stock solution (g/L)	
$NaNO_3$	100		15.0
K_2HPO_4	10		4.0
MgSO4·7H_2O	10		7.5
$CaCl_2·2H_2O$	10		3.6
Citric acid	10		0.6
Ferric ammonium citrate	10		0.6
$EDTANa_2$	10		0.1
Na_2CO_3	10		2.0
Trace metal solution	1	H_3BO_3	2.86
		$MnCl_2·4H_2O$	1.86
		$ZnSO_4·7H_2O$	0.22
		$Na_2MoO_4·2H_2O$	0.39
		$CuSO_4·5\ H_2O$	0.08
		$Co(NO_3)_2·6\ H_2O$	0.05

Figure S1. The settling characteristics of the different concentrations nano-TiO_2 with time.

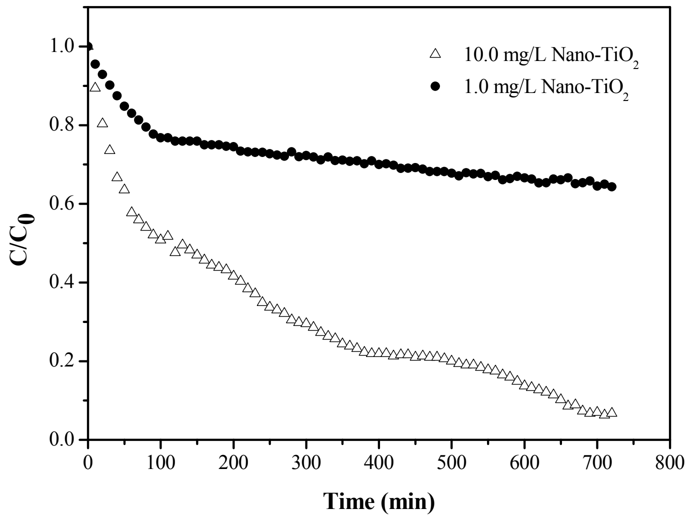

© 2013 by the authors; licensee MDPI, Basel, Switzerland. This article is an open access article distributed under the terms and conditions of the Creative Commons Attribution license (http://creativecommons.org/licenses/by/3.0/).
